# Assessment of Central Sleep Apnea Events in Children with Sleep-Disordered Breathing

**DOI:** 10.1155/2022/2590337

**Published:** 2022-05-17

**Authors:** Alyson Kaplan, Seckin O. Ulualp

**Affiliations:** ^1^Department of Otolaryngology-Head and Neck Surgery, University of Texas Southwestern Medical Center, Dallas, Texas, USA; ^2^Division of Pediatric Otolaryngology, Children's Health Dallas, Dallas, Texas, USA

## Abstract

**Purpose:**

To determine the prevalence of central apnea (CA) events and central sleep apnea (CSA) in children with sleep-disordered breathing (SDB) and to assess the effect of tonsillectomy and adenoidectomy (TA) on CSA in children with obstructive sleep apnea (OSA). *Material and Methods*. The medical charts of children with SDB were reviewed to obtain information on past medical history, polysomnography (PSG) findings, and surgical management. Counts and indexes of obstructive apnea, obstructive hypopnea, and central apnea were evaluated before and after TA. The prevalence of CSA and the effect of age, gender, obesity, and comorbid conditions on CSA were assessed in children with SDB as well as in children with PSG proven OSA.

**Results:**

Seven hundred twelve children with SDB (age range: 1 to 18 yrs, mean: 5.8 ± 3.4) were identified. CA events occurred in 640 of 712 (89.5%) patients. Of the 712 patients, 315 (44.2%) met the criteria for the diagnosis of CSA. CSA was more prevalent in toddlers and preschoolers (*p* < 0.001). Obese children had a higher prevalence of CSA compared to nonobese children (*p* < 0.001). The prevalence of CSA in patients with OSA was 45.4%. The number of CA events, CAI, and OAHI after TA was less than that of before TA (*p* < 0.001). Residual CSA after TA occurred in 20 children (26%).

**Conclusion:**

Central apnea events and central sleep apnea occur in children who present to a pediatric otolaryngology clinic for evaluation of sleep disordered breathing. Central sleep apnea and obstructive sleep apnea both improve after tonsillectomy and adenoidectomy.

## 1. Introduction

Pediatric sleep-disordered breathing (SDB) may be a manifesting presentation of sleep apnea, which may be classified as either obstructive or central in etiology [1]. Obstructive sleep apnea (OSA) is characterized by repetitive obstruction of the upper airway during sleep leading to reduction or complete cessation of breathing despite increased respiratory effort. In contrast, central sleep apnea (CSA) is defined by cessation of airflow without obstruction or evidence of respiratory effort. To date, OSA has been widely investigated in children with tonsillectomy and adenoidectomy surgery as a common and efficacious first-line treatment option; however, the characteristics of CSA in children who present to an otolaryngology clinic with SDB have not been systematically studied.

The prevalence of CSA in children ranged from 5.4% to 14.9% depending on the clinical setting, patient demographics, and criteria used to define CSA [2–4]. Potential clinical effects of CSA include altered heart rate and blood pressure [5]. Identification of the characteristics of CSA in children with SDB potentially improves the management of CSA and OSA in children. The aims of the present study are to determine the prevalence of central apnea events (CA) and CSA in children with SDB and to assess the effect of tonsillectomy and adenoidectomy (TA) on CSA in children with OSA.

## 2. Materials and Methods

The study was approved by the local institutional human research review board, and informed consent was waived. The charts of patients who were seen in a pediatric otolaryngology clinic between January 2009 and January 2019 were reviewed retrospectively. Patients seen by the senior author (S.O.U.) were identified using an electronic medical record. Patients who were under the age of 18 years and underwent polysomnography (PSG) to evaluate sleep-related breathing disorder were included in the study. Exclusion criteria included congenital central hypoventilation syndrome, late onset central hypoventilation syndrome, and a history of upper airway surgery including, but not limited to, tonsillectomy, adenoidectomy, palatoplasty, pharyngoplasty, turbinate reduction, septoplasty, supraglottoplasty, and tracheostomy. Upper airway surgery alters the airway patency; therefore, history of upper airway surgery was considered as an exclusion criterion. Patients were not excluded due to craniofacial anomalies, developmental delay, psychiatric illness, immunodeficiency, possible neoplasia, possible posttransplant lymphoproliferative disorder, or other chronic conditions.

Indications to obtain PSG were uncertainty about the need for surgery, discordance between tonsillar size on physical examination and the reported severity of SDB, inadequate description of sleep-related breathing disorder symptoms by caregivers, caregivers desire to avoid surgery, and the presence of complex medical conditions including but not limited to obesity, Down syndrome, craniofacial abnormalities, neuromuscular disorders, sickle cell disease, or mucopolysaccharidoses. Indications to obtain postoperative PSG were the persistent SDB symptoms or caregiver concerns, presence of severe OSA prior to TA, the presence of complex medical conditions including but not limited to obesity, Down syndrome, craniofacial abnormalities, neuromuscular disorders, sickle cell disease, or mucopolysaccharidoses. All patients had an all-night attended PSG in a sleep laboratory of a tertiary care children's hospital. Pediatricians board-certified in sleep medicine interpreted all PSGs. Respiratory effort was measured by dual thoracic, and abdominal respiratory inductance plethysmography. The criteria of the American Academy of Sleep Medicine were used to determine sleep measurements. The obstructive apnea-hypopnea index (OAHI) was calculated as the sum of obstructive apnea and hypopnea (OAH) per hour. The central apnea index (CAI) was calculated as central apnea (CA) per hour. The severity of CSA and OSA was categorized according to CAI and OAHI: mild, between 1 and 5 events/hr; moderate, between 5 and 10 events/hr; or severe, greater than 10 events/hr.

The following data were collected: age, gender, body mass index (BMI) percentile, past medical history, and PSG findings including counts and indexes of obstructive apnea, obstructive hypopnea, and central apnea. PSG findings before and after TA were included when available. BMI percentile was calculated according to Centers for Disease Control and Prevention growth standards. Patients were grouped into obese and nonobese based on the BMI percentile. The obese group included children with a BMI greater than the 95th percentile. The effect of age on airway obstruction findings for the following age groups was determined: toddler (1-3 years), preschooler (3-5 years), middle childhood (6-11 years), young teens (12-14 years), and teenager (15-18 years [6]. The effects of age, obesity, comorbidity, and severity of OSA on the CA and CAI were assessed.

Statistical comparisons between pre- and post-TA PSG findings were performed using parametric (one-way analysis of variance) and nonparametric tests (Kruskal Wallis one-way analysis of variance) as appropriate. Dunn's method was used to identify which group or groups differed from the others. Comparisons of prevalence were performed by a chi-square test. Pearson correlation calculated correlation. A *p* value less than 0.05 was deemed statistically significant. Results included odds ratio (OR) with 95% confidence interval (CI). Data are presented as mean ± standard deviation.

## 3. Results

Seven hundred twelve children with SDB (age range: 1 to 18 yrs, mean: 5.8 ± 3.4) underwent PSG (Table 1). Central apnea (CA) events occurred in 640 of 712 (89.5%) patients. Of the 712 patients, 315 (44.2%) met the criteria for the diagnosis of CSA. The severity of CSA was mild in 285 patients, moderate in 25, and severe in 5. OAH events occurred in 692 of 712 patients (97%). Of the 712 patients, 602 (84.5%) met the criteria for the diagnosis of OSA. The severity of OSA was mild in 179 patients, moderate in 122, and severe OSA in 301. CA, CSA, and OAHI were similar between male and female children (*p* = 0.6) (Table 1). Obese children had similar CA and CSA compared to nonobese children (*p* = 0.4). OAHI in obese children was higher than that of nonobese children (95% CI: 0.2, 8.6; *p* = 0.005). Toddlers had a higher CA and CSA than middle childhood children (95% CI: 1.47, 6.33; 95% CI: 0.02, 0.87, respectively) and teenagers (95% CI: 2.74, 11.75; 95% CI: 0.33, 1.63, respectively) (*p* < 0.001). Similarly, preschoolers had a higher CA and CSA than middle childhood children (95% CI: 0.03, 3.6; 95% CI: 0.29, 0.49, respectively) and teenagers (95% CI: 2.40, 7.5; 95% CI: 0.17, 1.04, respectively) (*p* < 0.001). CA and CSA were similar between toddlers and preschoolers (*p* = 1). OAHI was similar amongst the studied age groups (*p* > 0.05).

**Figure 1:**
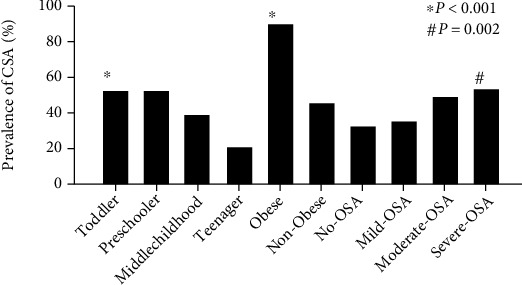
The prevalence of CSA amongst the studied age, weight, and OSA categories is shown. CSA was more prevalent in toddlers and preschoolers (*p* < 0.001). Obese children had a higher prevalence of CSA compared to nonobese children (*p* < 0.001). The prevalence of CSA in patients with moderate OSA and severe OSA was higher than that of patients with no OSA and mild OSA (*p* = 0.002).

**Figure 2:**
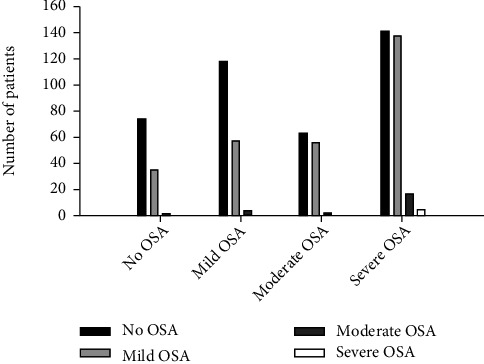
Distribution of central sleep apnea severity in children with sleep disordered breathing is shown. Severity of central sleep apnea (CSA) varied among the patients with no obstructive sleep apnea (OSA), mild OSA, moderate OSA, and severe OSA. Children with no OSA, mild OSA, or moderate OSA did not have severe CSA. Severe CSA occurred in children with severe OSA.

**Figure 3:**
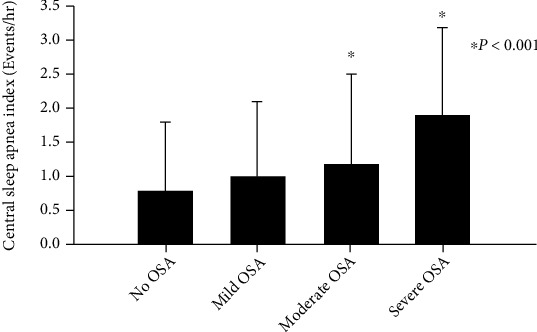
Comparison of central apnea index (CAI) in children with obstructive sleep apnea (OSA). The CAI in patients with severe OSA was higher than that of patients with no OSA, mild OSA, and moderate OSA (*p* = 0.001).

The prevalence of CSA amongst the studied age, weight, and OSA categories are shown in Figure 1. CSA was more prevalent in toddlers compared to middle childhood (OR: 1.7, 95% CI: 1.18, 2.45) and teenager age groups (OR: 4.1, 95% CI: 2.07, 8.23) (*p* < 0.001). Prevalence of CSA in preschoolers was higher than middle childhood (OR: 1.68, 95% CI: 1.14, 2.47) and teenager age groups (OR: 4.06, 95% CI: 2.01, 8.20) (*p* < 0.001). Obese children had a higher prevalence of CSA compared to nonobese children (OR: 9.69, 95% CI: 6.12, 15.36) (*p* < 0.001). The prevalence of CSA in patients with OSA was 45.4%. The prevalence of CSA in patients with moderate OSA was higher than patients with no OSA (OR: 1.96, 95% CI: 1.10, 3.48) and mild OSA (OR: 1.79, 95% CI: 1.01, 3.16) (*p* = 0.002). CSA was more prevalent in patients with severe OSA than patients with no OSA (OR: 2.39, 95% CI: 1.34, 4.25) and mild OSA (OR: 2.18, 95% CI: 1.23, 3.87) (*p* = 0.002). Severity of CSA varied among the patients with no OSA, mild OSA, moderate OSA, and severe OSA (Figure 2). Children with no OSA, mild OSA, or moderate OSA did not have severe CSA. Severe CSA occurred only in children with severe OSA. The CAI in patients with severe OSA was higher than that of patients with no OSA (95% CI: 0.45, 1.66), mild OSA (95% CI: 0.43, 1.37), and moderate OSA (95% CI: 0.05, 1.19) (*p* = 0.001) (Figure 3). A significant positive correlation was not found between the CAI and OAHI with an *r* value of 0.2 (*p* < 0.05).

One hundred forty-seven patients (86 male, 61 female, age range: 1 to 17 yrs, mean: 5.6 ± 3.3) had PSG before and after TA (Table 2). Seventy-eight children had CSA. The severity of CSA was mild in 64 patients, moderate in 12, and severe in 2. In patients with CSA, the severity of OSA before TA was mild in 1 patient, moderate in 10, and severe in 67. CAI was reduced in all patients after TA. Residual CSA occurred in 20 children (26%). Of the 20 patients with residual CSA after TA, 17 patients had severe OSA, and 3 had moderate OSA before TA. The prevalence of residual CSA was 25% in patients with severe OSA and 30% in patients with moderate OSA.

Of the 64 patients with mild CSA, 17 (26%) patients continued to have mild CSA after TA. In 12 patients with moderate CSA, moderate CSA persisted in 1 patient and diminished to mild CSA in 2 patients. In two patients with severe CSA, CSA was resolved in 1 patient and diminished to moderate CSA in 1. The number of CA events (95% CI: 3.11, 8.35), CAI (95% CI: 0.59, 1.49), and OAHI (95% CI: 7.24, 14.21) after TA was less than that of before TA (*p* < 0.001) (Table 2). Male and female children had less CA events (male, 95% CI: 3.11, 8.35; female, 95% CI: 2.53, 11.31) CAI (male, 95% CI: 0.59, 1.49; female, 95% CI: 0.64, 2.18), and OAHI (male, 95% CI: 18.09, 26.49; female, 95% CI: 20.69, 38.44) after TA (*p* < 0.001) (Table 2). The toddler and middle childhood age groups had less number of CA events (toddler, 95% CI: 3.47, 13.97; middle childhood, 95% CI: 2.90, 8.73) and CAI (toddler, 95% CI: 0.77, 2.31; middle childhood 95% CI: 0.53, 1.88) after TA (*p* < 0.001) (Table 2). The preschooler and teenager age groups did not have less number of CA events and CAI after TA (*p* = 0.1 and *p* = 0.2) (Table 2). OAHI after TA was less than that of before TA in toddler (95% CI: 21.2, 36.9), preschooler (95% CI: 12.79, 32.46), middle childhood (95% CI: 16.16, 29.68), and teenager (95% CI: 4.22, 47.19) (*p* < 0.001) (Table 2).

Thirty-one of the 147 patients who underwent TA had a comorbid condition: Down syndrome in 7 patients, seizure in 6, gastroesophageal reflux in 6, developmental delay in 3, hypothyroidism in 3, attention deficit hyperactivity disorder in 2, macrocephaly in 2, Jeune syndrome in 1, and autism in 1. Prior to TA, CA events, CAI, and OAHI in patients with comorbid conditions were not different than that of patients without comorbid conditions (*p* > 0.05) (Table 2). In patients with comorbid conditions, CA events and CAI before and after TA was not significantly different (*p* = 0.3). After TA, OAHI was less than before TA in children with comorbid conditions (95% CI: 12.65, 27.78) (*p* < 0.001) (Table 2). In children without comorbid conditions, CA (95% CI: 3.54, 7.93), CAI (95% CI: 0.73, 1.59), and OAHI (95% CI: 21.42, 31.08) was less after TA (*p* < 0.001) (Table 2).

## 4. Discussion

Children with SDB are commonly referred to otolaryngologists to consider surgical options for the treatment of sleep abnormalities. Caregivers often report pauses in breathing which may occur due to central or obstructive apnea. PSG provides an objective assessment of the characteristics of SDB. Better understanding of the characteristics of CSA in children with SDB potentially improves the management of children with SDB.

In the present study, the majority of children with SDB had CA events. The high prevalence of CA events should be considered in the assessment of caregiver reported apnea or pauses in breathing as well as in the management of respiratory abnormalities encountered in peri- and postoperative management of children with SDB. Despite the high prevalence of CA events in children with SDB, CSA criteria was met in 44% of our patients with SDB. The prevalence of CSA was 45.4% in children with PSG proven OSA. The prevalence of CSA in children has been reported with varying rates depending on the characteristics of the studied patient groups and the criteria used to define CSA. The prevalence of CSA was 14.9% in children with OSA, 5.4% in children who was referred to a sleep laboratory, and 13.3% in children who were recruited from pulmonary medicine, pediatric, psychiatric, neurology, and otolaryngology clinics [2–4]. The prevalence of CSA was 64% in children with OSA who had undergone tonsillectomy and adenoidectomy [7]. The PSG criteria used to define CSA influence the rate of CSA. Conventionally, a CAI equal or greater than 1 event/hr is used to define CSA; however, a CAI ≥ 5/h has been suggested to define CSA as healthy children who had CAI ≤ 6 h in previous studies [8, 9]. The optimum CAI threshold needs to be determined to better identify clinically significant CSA in children with SDB.

The effect of aging on CSA events in children with SDB has not been reported; however, airway length and airway length normalized for height in boys and postpubertal children were greater than girls and prepubertal children [10]. Earlier studies reported higher CAI in healthy younger children and children with craniosynostosis [10–12]. In a group of children who were recruited from pulmonary medicine, pediatric, psychiatric, neurology, and otolaryngology clinics, the prevalence of CSA declined from 34.1% in toddlers, 19.3% in preschool age, and 8.9% in school age to 1.3% in adolescence [4]. Our findings documented a similar decline in the prevalence of CSA in children with SDB. The prevalence of CSA was higher in toddlers and preschoolers compared to middle childhood and teenagers. Consistent with previous studies, our findings provided additional evidence that the prevalence of CSA varies depending on the population studied [4, 10–13].

The effect of obesity on CSA has not been established. Obesity may lead to inhibition of the respiratory center via upper airway mechanoreceptor stimulation due to pharyngeal collapse or reduction in oxygen reserve due to reduced thoracic volume [14, 15]. For example, studies have shown that waist circumference and BMI *z*-score are useful in predicting CSA [14]. In one study, the average CAI in the obese group was significantly lower than that in the nonobese group, indicating that obesity may protect children from developing CSA [4]. On the other hand, obesity is associated with increased serum leptin level, which prevents respiratory depression by increasing CO_2_ chemosensitivity during sleep in obesity [16]. In the present study, CSA was more prevalent in SDB children who were obese; however, SDB children who were obese had similar CA and CAI compared with SDB children who are nonobese. Obesity's effect on the pathogenesis of CSA merits further investigation.

The effect of severity of OSA on the prevalence of CSA was documented in children with sleep SDB who aged 14 and younger [17]. Greater number of CSA occurred in children with OSA compared to children without OSA [17]. The percentage of children with OSA who had CSA increased with greater degrees of OSA [17]. Similar to findings of the previous study [17], present study findings showed a higher prevalence of CSA in children with moderate and severe OSA than children with mild OSA and no OSA. The CAI in children with severe OSA was higher than children with no OSA, mild OSA, and moderate OSA. Nevertheless, there was no significant positive correlation between the CAI and OAHI.

A previous study has reported the effect of TA on CSA in children with OSA [3, 7]. In a group of 15 children, CSA was resolved in 11 children (73.3%) after TA. While the majority of children (90%) with mild CSA had resolution of CSA after TA, 75% of children with CAI greater than 5 before TA had residual CSA following TA. In a single child, CSA was more severe after TA. In a group of children 41 children who had undergone, tonsillectomy, adenoidectomy, or adenotonsillectomy, postoperative CAI was significantly less than preoperative CAI [7]. In children with Down syndrome, CAI was normalized in 10 of the 15 children [18]. In the present study, CAI was reduced significantly after TA. Residual CSA occurred in 26% of children with OSA. The majority of our patients with CSA had mild CSA. The rate of residual CSA was 26% in children with mild CSA. The higher rate of residual CSA in our patients compared to an earlier report may be due to differences in patient characteristics such as age, comorbidities, obesity, number of patients, and CSA index criteria for residual CSA. The prevalence of residual CSA in children with moderate and severe OSA was 30% and 25%, respectively, although the small number of children in these categories is a limitation of the study. Postoperative PSG may be considered to evaluate for residual CSA in children with CSA as well as in children with moderate or severe OSA.

This study documents the frequent occurrence of central apnea events, high prevalence of CSA, the effect of age and obesity on CSA, and improvement in CSA after TA in a large group of children with SDB. CA events and CSA have the potential to worsen clinical symptoms in children with OSA. A detailed evaluation of sleep-related respiratory patterns should be performed in children with SDB. In a clinical setting, our findings of higher prevalence of CA events and CSA in toddlers, preschooler, and obese children may be used to consider obtaining PSG as well as to assess respiratory events in the immediate postoperative period and clinical surveillance period. The pathophysiologic mechanism that correlates CSA, SDB, and OSA should be systematically investigated to delineate the role of CSA in the clinical assessment of SDB and surgical management of children.

Selection bias was present in the present study and previous studies investigating CSA in children with SDB or OSA due to strict inclusion criteria. PSG was used to assess CSA and OSA in children included in the present study. Postoperative PSG was obtained in children who met the inclusion criteria. The results of previous studies as well as current study cannot be generalized to children with SDB who did not have PSG. Standardized questionnaires such as Pediatric Sleep Questionnaire can be used to evaluate SDB in conjunction with PSG [19, 20]. In the present study, PSG was used to obtain quantifiable assessment of CSA and OSA. Limitations of our study are due to the composition of the study groups, the criteria used to define CSA, and the unequal distribution of subjects in subgroups of CSA and OSA severity. The subject population included children who were seen in a pediatric otolaryngology clinic at a tertiary care center. Our patients may represent a group of children in whom severe disease is suspected. Therefore, our findings of high prevalence of CA events and CSA may not represent the prevalence of CA and CSA in a community-based sample of children with sleep-disordered breathing. American Academy of Otolaryngology-Head and Neck Surgery, American Academy of Pediatrics, and American Academy of Sleep Medicine published guidelines with varying recommendations to obtain PSG in children [21–23]. In the present study, PSG was obtained in accordance with American Academy of Otolaryngology-Head and Neck Surgery guidelines or when a caregiver requested a PSG. The limited number of children with mild OSA was due to the use of American Academy of Otolaryngology-Head and Neck Surgery guidelines potentially resulted in the unequal distribution of the subject in the mild, moderate, and severe OSA groups. We could not draw conclusions regarding the effect of TA on CSA in children with mild OSA due to the small number of children with mild OSA.

The mechanism behind CSA resolution after TA has not been ascertained. In a previous study application of a local anesthetic to the pharynx resulted in reduction of number of central apneas [24]. Plausibly, the effect of relief of upper airway obstruction on the upper airway mechanoreceptors inhibiting respiratory drive may result in the reduction in central apneas after TA in children [7]. Improved ventilatory control after TA has been suggested to lead to reduction in central apneas during sleep [3]. Another plausible explanation of improvement in CSA after TA suggested an increase in airway resistance may cause increased CO_2_ levels activating central chemoreceptors which increases upper airway relaxation and unstable muscle activity [25–27]. Future studies are needed to elucidate the mechanism behind CSA resolution after TA.

## 5. Conclusions

CA events and CSA occur in children who present to a pediatric otolaryngology clinic for evaluation of SDB. The prevalence of CSA is higher in toddlers, preschoolers, and obese children with SDB. Varying severity of CSA occurs in children with OSA. CSA is more prevalent in children with moderate and severe OSA. In this group of children, severe CSA occurred in children with severe OSA. CSA as well as OSA improves after TA.

## Figures and Tables

**Table 1:** Demographics and polysomnography findings.

Patient groups	CA	CAI	OAHI
Entire cohort (*N* = 712)	8.6 (0-121)	1.4 (0-31.9)	14.7 (0-142.2)
Gender			
Male (*N* = 406)	8.6 (0-115)	1.3 (0-15.4)	14.4 (0-141.3)
Female (*N* = 306)	8.7 (0-121)	1.4 (0-31.9)	15.0 (0-142.2)
Age group			
Toddler (*N* = 214)	11.1 (0-121)	1.7 (0-16.3)	16.9 (0-142.2)
Preschooler (*N* = 171)	8.9 (0-46)	1.3 (0-8.5)	12.4 (0-120.3)
Middle childhood (*N* = 268)	7.1 (0-50)	1.2 (0-31.9)	15.1 (0-117)
Teenager (*N* = 59)	3.9 (0-37)	0.7 (0-3.5)	15.4 (0-124.1)
Weight category			
Obese (*N* = 115)	8.0 (0-98)	1.3 (0-31.9)	18.4 (0-124.1)
Nonobese (*N* = 497)	8.9 (0-121)	1.5 (0-16.3)	14.2 (0-142.2)

Data presented mean (range); CA: central apnea events; CAI: central apnea index; OAH: obstructive apnea hypopnea events; OAHI: obstructive apnea hypopnea index.

**Table 2:** Comparison of pre- and postoperative polysomnography findings.

Patient groups	CA		CAI		OAHI	
	Pre-TA	Post-TA	Pre-TA	Post-TA	Pre-TA	Post-TA
Entire cohort (*N* = 147)	11.6 (1-121)^†^	5.4 (0-41)	1.9 (0.1-16.3)^†^	0.7 (0-5.3)	28.6 (2.8-142.2)^†^	3.4 (0-35.6)
Gender						
Male (*N* = 87)	11.5 (1-47) ^†^	5.7 (0-41)	1.8 (0.1-9.8)^†^	0.8 (0-5.3)	26.2 (2.8-90.1)^†^	3.9 (0-35.6)
Female (*N* = 60)	11.8 (1-121)^†^	4.9 (0-22)	2.1 (0.1-16.3)^†^	0.7 (0-3.2)	32.1 (2.2-142.2)^†^	2.5 (0-12.2)
Age group						
Toddler (*N* = 52)	15.3 (1-121)^†^	6.6 (0-38)	2.4 (0.1-16.3)^†^	0.9 (0-4.5)	31.1 (2.2-142.2)^†^	2 (0-11.5)
Preschooler (*N* = 28)	8.1 (1-46)	5.3 (0-24)	1.2 (0.1-6.9)	0.7 (0-3.2)	26.1 (2.3-121.1)^†^	3.5 (0.1-20)
Middle childhood (*N* = 58)	10.5 (1-47)^†^	4.6 (0-41)	1.9 (0.1-13)^†^	0.7 (0-5.3)	26.7 (2.8-127.5)^†^	3.8 (0-35.6)
Teenager (*N* = 9)	8.5 (1-21)	3.8 (0-18)	1.6 (0.1-6.1)	0.5 (0-2.6)	33.7 (10.8-90.1)^†^	7.9 (2.1-16)
Weight category						
Obese (*N* = 66)	10.4 (1-46)^†^	5 (0-38)	1.9 (0.1-13)^†^	0.7 (0-4.5)	27.9 (2.2-142.2)^†^	4. 5 (0-35.6)
Nonobese (*N* = 81)	10.2 (1-47)^†^	5.5 (0-41)	1.7 (0.1-9.8)^†^	0.8 (0-5.3)	28 (2.8-141.9)^†^	2.4 (0-28.2)
Comorbid condition						
Present (*N* = 31)	9.2 (1-46)	5.6 (1-38)	1.5 (0.1-9.8)	0.9 (0-4.5)	17.7 (2.8-48)^†^	5.3 (0-35.6)
Absent (*N* = 116)	10.5 (1-47) ^†^	5.1 (0-41)	1.8 (0.1-13)^†^	0.7 (0-5.3)	31 (2.3-142.2)^†^	2.8 (0-16)

Data presented mean (range); CA: central apnea events; CAI: central apnea index; OAH: obstructive apnea hypopnea events; OAHI: obstructive apnea hypopnea index; ^†^*p* < 0.001.

## Data Availability

Data are available on request through the corresponding author.
